# A Cross-Sectional Study on the Impact of Emergency Department Boarding and Crowding on Emergency Department Observation Unit Patient Populations

**DOI:** 10.1016/j.acepjo.2026.100363

**Published:** 2026-04-06

**Authors:** Kian D. Samadian, Charlotte W. Croteau, Melissa A. Meeker, Joshua J. Baugh

**Affiliations:** Department of Emergency Medicine, Massachusetts General Hospital, Harvard Medical School, Boston, Massachusetts, USA

**Keywords:** emergency department observation, boarding, patient throughput, resource utilization, capacity management, decision making

## Abstract

**Objectives:**

Emergency Department (ED) boarding affects patient flow and clinical decision making, but its impact on ED observation unit (EDOU) utilization remains unclear. In this study, we examine whether ED boarding census levels are associated with changes in the characteristics, care processes, and outcomes of patients placed in the EDOU.

**Methods:**

We performed a cross-sectional study of all adult EDOU encounters from July 1, 2023 to June 30, 2024 in a single academic ED (∼120,000 annual visits) with a 31-bed EDOU staffed by ED clinicians. Encounters were stratified into quartiles of ED boarding census (number of admitted patients boarding ≥2 hours). Patient demographics, encounter characteristics, resource utilization, and outcomes were compared using χ^2^ and Kruskal-Wallis tests.

**Results:**

Among 8,469 encounters, patient characteristics remained largely consistent across boarding quartiles. Most diagnostic testing and imaging did not vary significantly. Higher boarding levels were associated with longer ED-to-EDOU times and modest increases in specialty consultations, ultrasounds, and echocardiograms. Admission rates were slightly higher in the highest boarding quartile compared with the lowest. EDOU length of stay increased incrementally with higher boarding levels.

**Conclusions:**

ED boarding levels were not strongly associated with changes in EDOU patient characteristics, management, or outcomes. Structured eligibility criteria and dedicated review processes likely insulated EDOU use from short-term operational pressures. Modest increases in consultations, diagnostics, and admissions suggest that slightly more complex patients may be placed in the EDOU during periods of high boarding. Overall, EDOU function remained relatively stable, supporting its role as a consistent throughput mechanism even during ED crowding.

**The Bottom Line**

Does emergency department (ED) boarding change how observation units are used? In this single-center study of 8,469 observation unit encounters, higher boarding levels were not associated with major changes in who was placed in the observation unit or how they were managed. Patient demographics and most diagnostic testing remained stable across boarding levels. Higher boarding was linked to longer wait times to reach the observation unit, slightly longer observation stays, and small increases in consultations and inpatient admissions. Overall, observation unit use remained remarkably consistent, suggesting that structured eligibility criteria and review processes may help preserve observation unit function even during periods of severe ED crowding.

## 1 Introduction

### 1.1 Background

Emergency department (ED) boarding and crowding have proven to be persistent and complex challenges in emergency medicine in past decades.^1^^,^^2^ Boarding, defined as the time a patient remains in the ED after the decision to admit, has been associated with delays in care, increased patient morbidity, reduced staff satisfaction, and higher health care costs.^3^, ^4^, ^5^, ^6^, ^7^, ^8^ In response to rising ED volumes and constrained inpatient capacity, some research has examined how ED clinicians modify care decisions under strain, including lowering thresholds for ED-to-inpatient admissions and adjusting disposition planning.^9^, ^10^, ^11^, ^12^

### 1.2 Importance

However, little is known about whether these environmental pressures influence the use of ED Observation Units (EDOUs). EDOUs are designed to provide focused, time-limited evaluation and management for patients who require further assessment beyond an initial ED visit but may not need full inpatient admission.^13^ The EDOU serves both as a clinical care setting and an operational tool to manage ED throughput. However, the degree to which EDOU utilization is affected by ED operational capacity strain, characterized by high ED boarding burden and constrained availability of inpatient beds, is unknown. On one hand, prior studies suggest that crowding can lead to shifts in decision making throughout the ED regarding patient disposition.^14^, ^15^, ^16^ On the other, EDOU placement often follows structured criteria with clearly defined inclusion and exclusion criteria, and may involve additional oversight by observation-dedicated clinicians.^17^ These features could buffer the EDOU from short-term operational pressures, particularly if there is limited excess capacity or if eligibility criteria are tightly followed. During periods of intense crowding and limited inpatient bed availability, clinicians may face incentives to favor EDOU placement over inpatient admission for borderline patients, as observation status can offer continued monitoring and diagnostic evaluation while alleviating ED bed capacity. Although this strategy may confer short-term operational advantages, it may be inappropriate for patients who ultimately require a higher level of inpatient care. On the other hand, crowding can also restrict EDOU capacity and could plausibly make some clinicians more reticent to place higher-risk patients into time-limited observation pathways. Therefore, the impact of operational stress on EDOUs is difficult to predict based on intuition alone.

### 1.3 Goals of This Investigation

In this study, we examine whether ED boarding census levels are associated with changes in the characteristics, patterns of clinical resource utilization and disposition, and operational outcomes of patients placed in the EDOU. We hypothesize that higher boarding levels may be associated with changes in EDOU utilization, including increased diagnostic testing, longer ED lengths of stay (LOS), and higher rates of ultimate inpatient admission. By analyzing EDOU encounters across quartiles of ED boarder census, we aim to explore whether clinician decision making about EDOU disposition varies under different conditions of operational strain.

## 2 Methods

### 2.1 Study Design and Setting

We performed a cross-sectional study of all EDOU encounters of patients 18 years or older in a single urban academic emergency department from July 1, 2023, to June 30, 2024. During the study period, this ED saw approximately 120,000 patient visits per year and had a 31-bed EDOU staffed entirely by ED advanced practice providers (APPs). EDOU patient candidates were evaluated against inclusion and exclusion criteria, with placement decisions requiring approval by an ED observation APP and resource nurse. Patients appropriate for EDOU were anticipated to be discharged within 48 hours. This EDOU functioned as a Type 2 EDOU, where these APPs provided discretionary care rather than being guided by a predetermined care protocol.^18^ We excluded encounters in which a psychiatric consultation was ordered (as these patients were often in observation status in our setting, but do not travel to the EDOU) and encounters in which the ED disposition indicated that the patient left against medical advice or was transferred to another hospital. The study protocol was approved by our local Institutional Review Board.

### 2.2 Measurements and Outcomes

All patient and encounter characteristics were extracted from EPIC, the health system’s electronic health record. Extracted patient characteristics included age (18-64 years; >65 years), biological sex (male; female), race/ethnicity (Hispanic; non-Hispanic Black; non-Hispanic White; non-Hispanic other; unknown), and primary language (English; Spanish; other; unknown). Encounter characteristics included insurance (private, public, other, unknown), acuity as defined by the assigned emergency severity index (ESI) (1 to 2, 3 to 5, unknown), and various time intervals. Time from ED arrival to EDOU order was defined as the interval (in minutes) from patient presentation to the ED to the clinical decision of placing the patient in the EDOU. Time from EDOU order to EDOU arrival represented the interval (in minutes) required for EDOU eligibility review and operational approval by the observation advanced practice provider and resource nurse prior to physical transfer. Time from ED arrival to EDOU arrival reflected the total elapsed time (in minutes) from ED presentation to physical placement in the EDOU. ED LOS was defined as the time (in hours) from ED arrival to final disposition from the EDOU (discharge or inpatient admission). We also measured ED disposition (inpatient admission or discharge) and quantity of specific orders placed before and after the EDOU order. Specifically, we extracted the occurrence of orders for specialty consultations, intravenous (IV) access, telemetry/pulse oximetry, x-rays (XR), computed tomography (CT) scans, magnetic resonance imaging (MRI), all ultrasound imaging (US), and specifically echocardiograms (TTE), electrocardiograms (ECG), and quantity of lab tests. Orders were used as a proxy for patient case complexity rather than resource utilization. For all EDOU encounters, we quantified ED boarder census as the count of all ED boarders (patients with admission orders and boarding for ≥2 hours) at the time the encounter’s EDOU order was placed.

To evaluate variation across differing levels of ED boarding, ED boarder census was categorized into quartiles based on the empirical distribution of boarding burden at the time of EDOU order placement in our study cohort. Quartiles were used as a descriptive stratification approach to ensure approximately equal numbers of encounters within each category, thereby facilitating stable comparisons across a spectrum of operational states. Because quartiles reflect the observed distribution of boarding rather than evenly spaced numeric intervals, the resulting ranges were ≤30, 31-38, 39-46, and ≥47 boarders. This approach was selected to characterize how EDOU utilization and management varied across relative levels of operational strain within the observed practice environment, rather than to identify absolute capacity thresholds. As a result, quartiles should be interpreted as comparisons across progressively higher boarding burden states, not as discrete operational cutoffs. To further improve interpretability and contextual relevance, these values correspond to approximately ≤37%, 38-47%, 48-57%, and ≥58% of total ED bed capacity filled by ED boarders, respectively, based on this ED that had 81 treatment rooms. Notably, boarding levels corresponding to approximately ≥58% of ED bed capacity coincide with internal operational escalation thresholds at this institution, at which point system-level strategies, such as placement of admitted patients in inpatient hallway locations, are implemented.

### 2.3 Descriptive analysis of all variables overall and stratified by ED boarder census quartile

We presented the descriptive analysis as frequency/percent for categoric variables and median/interquartile range (IQR) for continuous variables. We then evaluated bivariate associations across all ED boarder census quartiles and all other patient and encounter characteristics with χ2 tests for categoric variables and Kruskal-Wallis tests for continuous variables. As a secondary analysis, we also evaluated bivariate associations between the lowest and highest ED boarder census quartiles for all patient and encounter characteristics.

## 3 Results

A total of 8,469 EDOU encounters were analyzed between July 1, 2023, and June 30, 2024. The mean EDOU daily census for this 31-bed unit was 27 patients, which translates to a mean occupancy rate of 87%. Encounters were stratified into quartiles based on the ED boarder census at the time of the EDOU disposition order: Quartile 1 (≤30 boarders), quartile 2 (31–38), quartile 3 (39–46), and quartile 4 (≥47), corresponding to ≤37%, 38–47%, 48–57%, and ≥58% of total ED bed capacity. Results for individual variables are depicted in Table 1, Table 2, Table 3, Table 4, Table 5, Table 6.

**Table 1:** Descriptive analysis of patient demographics in the EDOU, overall and stratified by boarding census quartile with results from bivariate statistical tests.

Patient encounter demographic		Total (n = 8,469)	Boarding census (and % of ED beds occupied) at time of EDOU Order			
			Quartile 1 ≤30 (≤37%) (n = 1,890)	Quartile 2 31-38 (38%-47%) (n = 2,218)	Quartile 3 39-46 (48%-57%) (n = 2,107)	Quartile 4 ≥47 (≥58%) (n = 2,254)
Age (y) n (%)	18-64	4,055 (48)	884 (47)	1,059 (48)	1,048 (50)	1,064 (47)
	>65	4,414 (52)	1,006 (53)	1,159 (52)	1,059 (50)	1,190 (53)
Sex n (%)	Male	3,797 (45)	847 (45)	992 (45)	966 (46)	992 (44)
	Female	4,672 (55)	1,043 (55)	1,226 (55)	1,141 (54)	1,262 (56)
Race/ethnicity n (%)	Hispanic	1,173 (14)	262 (14)	306 (14)	283 (13)	322 (14)
	Non-Hispanic Black	818 (10)	169 (9)	224 (10)	217 (10)	208 (9)
	Non-Hispanic White	5,409 (64)	1,217 (64)	1,402 (63)	1,358 (64)	1,432 (64)
	Non-Hispanic Other	672 (8)	152 (8)	175 (8)	158 (7)	187 (8)
	Unknown	397 (5)	90 (5)	111 (5)	91 (4)	105 (5)
Language n (%)	English	7,073 (84)	1,575 (83)	1,857 (84)	1,786 (85)	1,855 (82)
	Spanish	708 (8)	153 (8)	193 (9)	166 (8)	196 (9)
	Other	686 (8)	162 (9)	167 (8)	155 (7)	202 (9)
Insurance type n (%)	Private	2,445 (29)	506 (27)	675 (30)	641 (30)	623 (28)
	Public	5,660 (67)	1,306 (69)	1,441 (65)	1,396 (66)	1,517 (67)
	Other	184 (2)	40 (2)	53 (2)	37 (2)	54 (2)
	Missing	180 (2)	38 (2)	49 (2)	33 (2)	60 (3)
Acuity (ESI) n (%)	1-2	3,049 (36)	713 (38)	816 (37)	719 (34)	801 (36)
	3-5	5,378 (64)	1,168 (62)	1,393 (63)	1,373 (65)	1,444 (64)
	Missing	42 (0)	9 (0)	9 (0)	15 (1)	9 (0)

ED, emergency department; EDOU, ED observation unit.

**Table 2:** Results of χ2 tests for categoric variables and Kruskal-Wallis tests for continuous variables used in the analysis of patient demographics in the EDOU.

Patient encounter demographic		Bivariate Association Across All Quartiles (*P*)	Bivariate Association Between Lowest (Q1) and Highest (Q4) Quartiles (*P*)
Age (y)	18-64	.233	.805
	>65		
Sex	Male	.682	.626
	Female		
Race/ethnicity	Hispanic	.927	.978
	Non-Hispanic Black		
	Non-Hispanic White		
	Non-Hispanic Other		
	Unknown		
Language	English	.313	.687
	Spanish		
	Other		
Insurance type	Private	.021^a^	.396
	Public		
	Other		
	Missing		
Acuity (ESI)	1 to 2	.174	.313
	3 to 5		
	Missing		

EDOU, ED observation unit.

a Statistically significant with *P* < .05.

**Table 3:** Descriptive analysis of operational metrics related to the EDOU, overall and stratified by boarding census quartile. Time intervals are reported as median (IQR). Because values are summarized at the cohort level, the median time from ED arrival to EDOU arrival does not equal the sum of the medians for preceding intervals, although these intervals are additive at the individual encounter level.

Operational metric		Overall (n = 8,469)	Boarding census (and % of ED beds occupied) at time of EDOU order			
			Quartile 1 ≤30 (≤37%) (n = 1,890)	Quartile 2 31-38 (38%-47%) (n = 2,218)	Quartile 3 39-46 (48%-57%) (n = 2,107)	Quartile 4 ≥47 (≥58%) (n = 2,254)
Time of arrival to time of EDOU order minutes (IQR)		320 (229)	310 (211)	313 (216)	319 (227)	341 (262)
Time of EDOU order to EDOU arrival minutes (IQR)		89 (84)	79 (63)	85 (76)	90 (96)	100 (119)
Time from ED arrival to EDOU arrival minutes (IQR)		434 (269)	408 (225)	422 (235)	439 (278)	473 (352)
ED length of stay hours (IQR)		28 (21)	27 (18)	28 (22)	28 (20)	30 (23)
Disposition n (%)	Admit	1,540 (18)	309 (16)	444 (20)	354 (17)	433 (19)
	Discharge	6,929 (82)	1,581 (84)	1,774 (80)	1,753 (83)	1,821 (81)

ED, emergency department; EDOU, ED observation unit.

**Table 4:** Results of χ2 tests for categoric variables and Kruskal-Wallis tests for continuous variables used in analysis of operational metrics related to the EDOU.

Operational metric		Bivariate association across all quartiles (*P*)	Bivariate association between lowest (Q1) and highest (Q4) quartiles (*P*)
Time of arrival to time of EDOU order		.001^a^	<.001^a^
Time of EDOU order to EDOU arrival		<.001^a^	<.001^a^
Time from ED arrival to EDOU arrival		<.001^a^	<.001^a^
ED length of stay		<.001^a^	<.001^a^
Disposition	Admit	.004^a^	.019^a^
	Discharge		

ED, emergency department; EDOU, ED observation unit.

a Statistically significant with *P* < .05.

**Table 5:** Descriptive analysis of orders placed before and after a decision was made to admit a patient to the EDOU, overall and stratified by boarding census quartile.

Order		Overall (n = 8,469)	Boarding census (and % of ED beds occupied) at time of EDOU order			
			Quartile 1≤30 (≤37%)(n = 1,890)	Quartile 231-38 (38%-47%)(n = 2,218)	Quartile 339-46 (48%-57%)(n = 2,107)	Quartile 4≥47 (≥58%)(n = 2,254)
Pre-EDOU order	Consult n (%)	2,987 (35)	648 (34)	774 (35)	766 (36)	799 (35)
	IV access n (%)	195 (2)	46 (2)	63 (3)	36 (2)	50 (2)
	Telemetry/Pulse Ox n (%)	2,656 (31)	578 (31)	695 (31)	665 (32)	718 (32)
	CXR n (%)	3,460 (41)	757 (40)	894 (40)	842 (40)	967 (43)
	Nonchest XR n (%)	1,433 (17)	310 (16)	382 (17)	340 (16)	401 (18)
	CTH n (%)	2,158 (25)	473 (25)	563 (25)	528 (25)	594 (26)
	CTPE n (%)	356 (4)	66 (3)	98 (4)	86 (4)	106 (5)
	CTAP n (%)	1,520 (18)	320 (17)	390 (18)	394 (19)	416 (18)
	Brain MRI n (%)	1,171 (14)	245 (13)	317 (14)	288 (14)	321 (14)
	Nonbrain MRI n (%)	1,008 (12)	195 (10)	271 (12)	267 (13%)	275 (12)
	US n (%)	598 (7)	134 (7)	167 (8)	138 (7%)	159 (7)
	POCUS n (%)	1,270 (15)	282 (15)	355 (16)	292 (14%)	341 (15)
	TTE n (%)	549 (6)	99 (5)	154 (7)	137 (7%)	159 (7)
	Labs placed median (IQR)	12 (8)	12 (8)	12 (8)	12 (8)	12 (10)
	ECG n (%)	4,945 (58)	1,091 (58)	1,292 (58)	1,234 (59%)	1,328 (59)
Post-EDOU order	Consult n (%)	2,605 (31)	527 (28)	688 (31)	655 (31%)	735 (33)
	IV Access n (%)	75 (1)	13 (1)	22 (1)	17 (1%)	23 (1)
	Telemetry/Pulse Ox n (%)	1,396 (16)	284 (15)	378 (17)	315 (15%)	419 (19)
	CXR n (%)	413 (5)	79 (4)	109 (5)	96 (5%)	129 (6)
	Nonchest XR n (%)	440 (5)	84 (4)	107 (5)	123 (6%)	126 (6)
	CTH n (%)	207 (2)	35 (2)	50 (2%)	55 (3%)	67 (3)
	CTPE n (%)	116 (1)	21 (1)	32 (1)	30 (1%)	33 (1)
	CTAP n (%)	246 (3)	50 (3%)	83 (4%)	44 (2%)	69 (3)
	Brain MRI n (%)	279 (3)	57 (3%)	83 (4%)	55 (3%)	84 (4)
	Nonbrain MRI n (%)	406 (5)	83 (4%)	101 (5%)	98 (5%)	124 (6)
	US n (%)	290 (3)	53 (3)	79 (4%)	71 (3%)	87 (4)
	POCUS n (%)	246 (3)	37 (2)	64 (3%)	59 (3%)	86 (4)
	TTE n (%)	524 (6)	89 (5)	150 (7%)	137 (7%)	148 (7)
	Labs placed median (IQR)	2 (5)	2 (4)	2 (4)	2 (4)	2 (6)
	ECG n (%)	815 (10)	175 (9)	217 (10)	211 (10)	212 (9)

CTAP, computed tomography abdomen/pelvis; CXR, chest x-ray; CTH, computed tomography head; CTPE, CT pulmonary embolism; ECG, electrocardiogram; ED, emergency department; EDOU, ED observation unit; IV, intravenous; MRI, magnetic resonance imaging; POCUS, point-of-care ultrasound; TTE, echocardiogram; US, ultrasound; XR, x-ray.

**Table 6:** Results of χ2 tests for categoric variables and Kruskal-Wallis tests for continuous variables used in the analysis of orders placed before and after a decision was made to admit a patient to the EDOU.

Order		Bivariate Association Across All Quartiles (*P*)	Bivariate Association Between Lowest (Q1) and Highest (Q4) Quartiles (*P*)
Pre-EDOU order	Consult	.561	.454
	IV access	.095	.722
	Telemetry/Pulse Ox	.843	.397
	CXR	.146	.069
	Nonchest XR	.450	.255
	CTH	.725	.349
	CTPE	.251	.062
	CTAP	.435	.216
	Brain MRI	.582	.251
	Nonbrain MRI	.106	.064
	US	.663	1.000
	POCUS	.267	.886
	TTE	.078	.019^a^
	Labs placed	.202	.048^a^
	ECG	.886	.457
Post-EDOU order	Consult	.011^a^	.001^a^
	IV access	.627	.327
	Telemetry/Pulse Ox	.003^a^	.003^a^
	CXR	.115	.028^a^
	Nonchest XR	.154	.109
	CTH	.112	.027^a^
	CTPE	.749	.390
	CTAP	.011^a^	.481
	Brain MRI	.099	.242
	Nonbrain MRI	.319	.118
	US	.304	.074
	POCUS	.005^a^	<.001^a^
	TTE	.026^a^	.013^a^
	Labs placed	.047^a^	.009^a^
	ECG	.838	.914

CTAP, CT abdomen/pelvis; CXR, chest x-ray; CTH, CT head; CTPE, CT pulmonary embolism; ECG, Electrocardiogram; EDOU, ED observation unit; IV, intravenous; MRI, magnetic resonance imaging; POCUS, Point-of-care ultrasound; TTE, Echocardiogram; US, Ultrasound; XR, x-ray.

a Statistically significant with *P* < .05.

Most demographic characteristics, including age, sex, race/ethnicity, and primary language, did not differ significantly across ED boarding quartiles, with the only exception being insurance status (Table 1 and 2). Median ED LOS increased modestly with higher ED boarding levels, though absolute differences between quartiles were small (Table 3). In quartile 1 (lowest boarding census), the median LOS was 27 hours. Quartiles 2 and 3 revealed median LOS of 28 hours each. In quartile 4 (highest boarding census), the median LOS was 30 hours. The Kruskal-Wallis test indicated that the distribution of ED LOS was significantly different across quartiles of boarding census (Table 4).

Most clinical management variables did not vary across quartiles; there were no significant differences in CT scans, MRIs, ultrasounds, EKGs, or other standard imaging and diagnostic orders placed prior to EDOU assignment (Tables 5 and 6). A few post-EDOU decision resource utilization metrics were significantly different across quartiles: number of specialty consultations, point-of-care ultrasounds (POCUS), TTEs, and ultimate admission rate. The magnitudes of difference between the lowest and highest quartiles for these variables were all relatively small, such as a 2% increase in TTEs. Otherwise, the majority of variables measured after the decision to place the patient in EDOU were not significantly different across quartiles.

## 4 Limitations

This study has several limitations. First, it was conducted at a single academic medical center with a specific EDOU model, including formalized eligibility criteria and a structured review process prior to patient placement. These findings may not apply to settings with more discretionary or variable EDOU workflows. Second, we stratified ED boarding levels into quartiles to allow for comparisons across a range of operational conditions. Although useful, this approach may miss more granular or nonlinear changes that occur only at extreme levels of crowding. Future studies could apply continuous boarding metrics or alternate modeling approaches to better capture dynamic effects. Third, although our findings did not show major differences in EDOU utilization across shifts with variable boarding, it is possible that ED clinicians adapt more to general trends over time rather than to daily fluctuations. For example, the EDOU population in 2025 may be more complex than in prior years (or decades) due to the broader, long-term impact of worsening ED crowding. Our design did not allow us to evaluate such long-term temporal shifts. Fourth, although our findings suggest that some orders did increase after EDOU disposition during high boarding periods, our study design limits our ability to draw firm conclusions about temporal causality. Orders may be entered at different points in the workflow depending on clinician practice patterns, documentation habits, or electronic health record system workflows. Finally, this EDOU did not use a strict protocol-driven care model at the time of this study, which prevented us from measuring protocol compliance and limited our ability to draw parallels between order number and resource utilization. Future studies using prospective observation and studying different EDOU care models could better clarify how care patterns evolve over time in response to crowding.

## 5 Discussion

In this study, we evaluated whether ED boarding levels influence the characteristics, management, and outcomes of patients placed in a large EDOU operated solely by ED clinicians. Although prior research has shown that ED clinicians may adjust admission decisions in response to operational pressures like crowding and boarding, in our study, we observed little change in EDOU utilization across varying levels of ED boarder census.^19^, ^20^, ^21^, ^22^ Most patient characteristics, acuity (ESI) levels, clinical management variables, and outcomes remained consistent across boarding quartiles; although there were significant differences in a few categories, including consultations, TTEs, EDOU LOS, and ultimate admission rate, the magnitude of these differences was mostly small and did not always follow a clear trend across all quartiles. Altogether, our results suggest that EDOU use may be less sensitive to short-term operational fluctuations than previously observed with inpatient admissions.^23^ The observed increase in time from ED arrival to EDOU arrival with higher levels of ED boarding likely reflects broader slowdowns in ED care processes during periods of crowding. However, once in the EDOU, it does not appear patients received different types of care based on levels of operational stress. This finding aligns with the interpretation that once patients are placed in the EDOU, care decisions remain relatively consistent.^24^, ^25^, ^26^

There are several possible reasons for these findings. Unlike inpatient admissions, EDOU placement at our institution was governed by a relatively structured workflow. During the study period, patient eligibility was guided by overarching EDOU criteria with defined exclusion criteria, and all candidates were reviewed by a resource nurse and ED observation APPs before being accepted. This gatekeeping process may have added stability to patient selection, reducing the influence of variable boarding pressure on EDOU decision making. Furthermore, EDOUs may be inherently more insulated from crowding effects than inpatient units because of their defined time-limited function, stronger control by ED clinicians, and a narrower range of appropriate patients. Finally, it is worth noting that our EDOU generally operated near or at capacity during the study period; an EDOU with routine excess capacity (ie, empty beds) might be more susceptible to operational pressures, such as crowding.

The exceptions to our overall null findings are worth noting: increases in consultations, echocardiograms, and POCUS orders after EDOU disposition, as well as modest but statistically significant increases in EDOU LOS and inpatient admission rates at higher boarding levels (of note, these findings were evaluated as bivariate associations and not adjusted for confounders). These findings, specifically the increase in specialty consultations, suggest that the patients placed in the EDOU during periods of high boarding may be slightly more complex than the average EDOU patient. Providers may be more willing to place borderline patients in the EDOU when inpatient beds are scarce, leading to modest increases in diagnostic intensity and LOS. This may also be due to the possible lengthy nature of specialty consultation. The presence of statistically significant differences only between the lowest and highest quartiles for some of these categories perhaps supports a potential threshold effect, where practice may shift only at the extremes of operational stress.^27^ These areas may be worth exploring in future research involving other EDOU contexts.

Overall, our findings suggest that although ED clinicians have been shown to adjust inpatient admission practices in response to crowding, they appear to maintain relatively more stable thresholds for EDOU placement during day-to-day fluctuations in boarding, at least in our particular EDOU context.

## Author Contributions

JJB, KDS, and CWC developed the manuscript concept. JJB, KDS, CWC, and MAM implemented the study design. MAM performed qualitative and quantitative data collection and analysis. KDS, JJB, MAM, and CWC all provided critical revisions of the manuscript for important intellectual content.

## Conflict of Interest

All authors have affirmed they have no conflicts of interest to declare.
